# Solid-Phase Parallel Synthesis of Drug-Like Artificial 2*H*-Benzopyran Libraries

**DOI:** 10.3390/molecules17055467

**Published:** 2012-05-09

**Authors:** Taeho Lee, Young-Dae Gong

**Affiliations:** 1 Research Institute of Pharmaceutical Sciences, College of Pharmacy, Kyungpook National University, 1370, Sangyuk-dong, Buk-gu, Daegu 702-701, Korea; Email: tlee@knu.ac.kr; 2 Center for Innovative Drug Library Research, Department of Chemistry, College of Natural Science, Dongguk University-Seoul, 26 Pildong 3-ga, Jung-gu, Seoul 100-715, Korea

**Keywords:** combinatorial chemistry, solid-phase synthesis, chemical library, drug-like molecules, 2*H*-benzopyran

## Abstract

This review covers the construction of drug-like 2*H*-benzopyrans and related libraries using solid-phase parallel synthesis. In this context, the preparation of substituted benzopyrans such as mono-, di- and trisubstituted benzopyran derivatives and additional ring-fused benzopyrans such as benzopyranoisoxazoles, benzopyranopyrazoles, six-membered ring-fused benzopyrans, and polycyclic benzopyrans are highlighted.

## 1. Introduction

Combinatorial chemistry has become an extremely powerful technique for the generation of drug-like and biologically active small organic molecule libraries in either the solution-phase or on solid supports [1,2,3,4,5]. In combinatorial synthesis, solid-phase organic synthesis (SPOS) is now routinely used to prepare a large number of small heterocyclic molecules and is especially useful in creating massive numbers of hit and lead compounds as part of high-throughput screening (HTS) technologies [6,7,8,9,10]. This is especially true for the privileged structures, which are core components of a large number of substances that possess a wide range of interesting biological activities and have been developed on solid-phase strategies [11,12,13,14,15].

**Figure 1:**
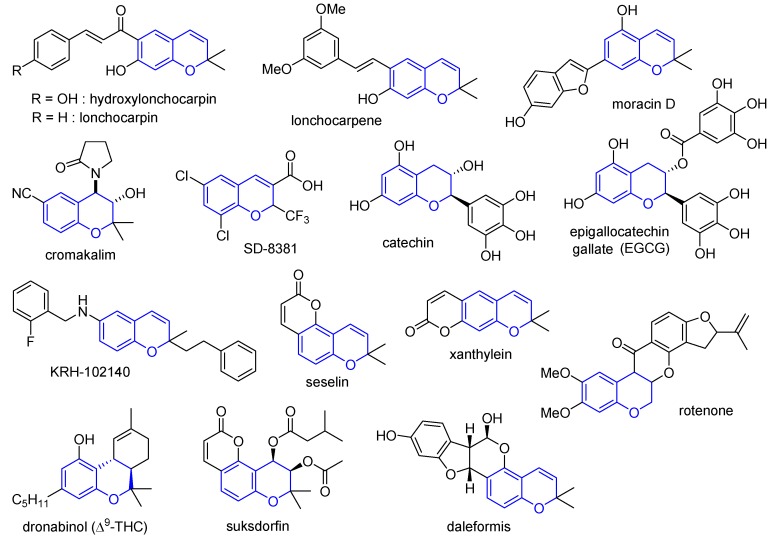
The representative examples of benzopyran-containing natural products and artificial bioactive molecules.

Among biologically active heterocyclic scaffolds, the well-known privileged benzopyran structure frequently appears in many natural products and artificial bioactive molecules, that exhibit a wide range of biological activities [16,17,18,19,20,21,22,23,24,25,26,27,28,29,30,31,32,33,34,35,36,37,38,39,40,41,42,43,44,45,46,47,48,49]. Representative examples of benzopyran-containing natural products and artificial bioactive molecules are illustrated in Figure 1.

Hydroxylonchocarpin, lonchocarpin, and lonchocarpene exhibit anticancer activities [16,17,18], whereas moracin D exhibits antifungal activities [19,20]. Cromakalim [21,22,23,24] with its benzopyran moiety produces anti-hypertensive effects via potassium channel modulation, and SD8381 [25,26] with a 2*H*-benzopyran scaffold represents anti-inflammatory effects as a novel cyclooxygenase (COX)-2 inhibitor. Catechin and epigallocatechin gallate (EGCG) show both an antiallergic effects and anticancer action [27,28,29], whereas KRH-102140 with a 2*H*-benzopyran moiety is identified as a 5-lipoxygenase (5-LO) inhibitor [30,31]. Also, seselin and xanthylein with tricyclic benzopyrans exhibit anticancer activities [32,33,34].

(−)-D^9^-Tetrahydrocannabinol ((−)-D^9^-THC, dronabinol) and other cannabinoids have been used to treat the symptoms of cancer, pain relief, and spasticity in multiple sclerosis, or as appetite stimulants for acquired immunodeficiency syndrome (AIDS) patients [35,36,37,38,39]. Suksdorfin (khellactone ester) inhibits human immunodeficiency virus (HIV)-1 replication in H9 lymphocytes [40,41], whereas daleformis shows inhibitory activities against endothelin-converting enzyme [42]. Rotenone has been used as an antianaphylactic agent for the treatment of asthma [43,44,45,46,47,48,49].

**Figure 2:**
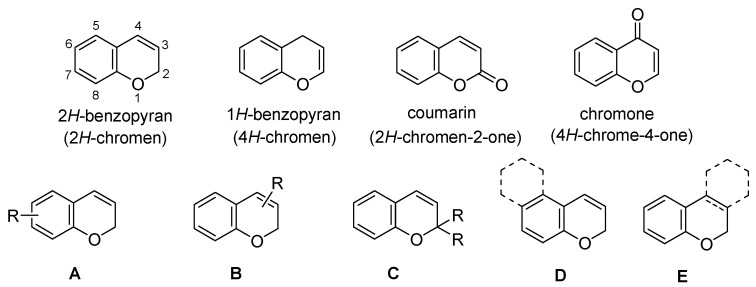
The core structures of benzopyran.

This paper reviews the use of solid-phase parallel synthesis in the construction of 2*H*-benzopyran libraries that contain substituted 2*H*-benzopyrans (**A**, **B**, and **C**) and additional cycle-fused benzopyrans (**D** and **E**), except the derivatives of 1*H*-benzopyran, coumarin, and chromone moieties (Figure 2). In addition, the solid-phase synthesis of modified benzopyran derivatives using the 2*H*-benzopyran moiety as an intermediate is also discussed. The sections have been divided according to the number of substituents in the benzopyran core and the kinds of fused-benzopyran cores. Publications cited herein are mostly refereed journals and not patents.

## 2. Solid-Phase Synthesis of Substituted Benzopyran Compounds

### 2.1. Solid-Phase Synthesis of Monosubstituted Benzopyran Compounds

#### 2.1.1. Solid-Phase Synthesis of 3-Substituted Benzopyran Compounds

**Scheme 1:**
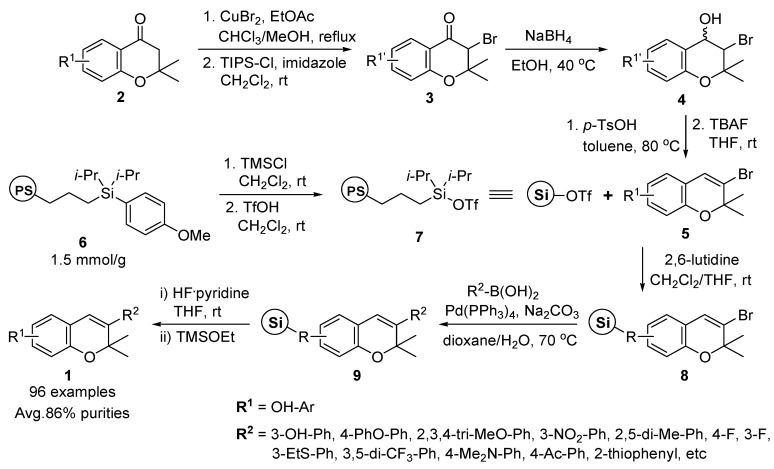
Solid-phase synthesis of 3-substituted 2*H*-benzopyrans **1** by Park *et al.* [50].

Park and co-workers reported the solid-phase synthesis of 3-substituted 2*H*-benzopyran **1** (Scheme 1) and the fluorous-tag-based solution-phase synthesis of benzopyran derivatives with discrete core scaffolds to construct a 284-member library [50].

**Figure 3:**
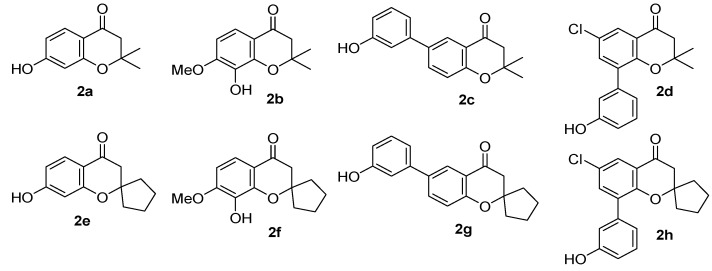
Various structures of 2*H*-benzopyran precursor (chromanone) moieties **2**.

The library construction was started from four different chromanones **2** (**2a**–**d**, see Figure 3), which were subjected to α-bromination and subsequent silyl protection at the phenolic hydroxyl group. α-Bromoketones **3** were reduced to α-bromoalcohols **4** by NaBH_4_, followed by acid-catalyzed dehydration and subsequent silyl deprotection, to yield four vinyl bromides containing 2*H*-benzopyran moiety **5**. After the activation of (4-methoxyphenyl)-diisopropylsilylpropyl polystyrene resin **6** with TfOH, vinyl bromide intermediates **5** were immobilized on the activated resin **7** in the presence of 2,6-lutidine to afford polymer-bound intermediates **8**.

The 3-substituted 2*H*-benzopyran resins **9** were introduced to various aryl and heteroaryl moieties via Suzuki coupling of boronic acids (24 commercially available aryl- and heteroaryl-boronic acids) with Pd(PPh_3_)_4_ and Na_2_CO_3_ in aqueous 1,4-dioxane with high yields and purity. After the standard cleavage protocol of silyl linkers using HF/pyridine and subsequent quenching with TMSOEt, the desired 3-substituted 2*H*-benzopyrans **1** (96 examples) were successfully prepared on a scale of 5–10 mg with an average purity of 86%.

#### 2.1.2. Solid-Phase Synthesis of 4-Substituted Benzopyran Compounds

**Scheme 2:**
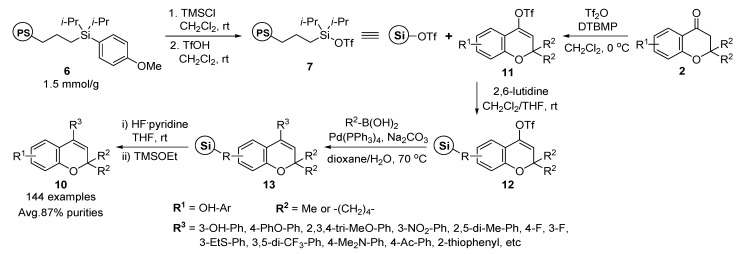
Solid-phase synthesis of 4-substituted 2*H*-benzopyrans **10** by Park *et al.* [51].

Park and co-workers also reported the solid-phase synthesis of 4-substituted 2*H*-benzopyran **10** using similar methods for the solid-phase synthesis of 3-substituted 2*H*-benzopyran **1** (see Scheme 1) except 3-triflated moiety in **11** as starting materials instead of 3-bromo-benzopyrans **5** (Scheme 2) [51].

The synthetic route for 4-substituted 2*H*-benzopyran **10** was initiated from triflate resins **12**, which were derived from eight different 2*H*-benzopyran moieties **2** (**2a**–**h**, see Figure 3), and the activated solid support **7**. 4-Triflate-benzopyrans **11** were synthesized from four different hydroxyacetophenones through cyclization with acetone [R^2^ = methyl] or cyclopentanone [R^2^ = –(CH2)4–] in the presence of pyrrolidine catalyst, and triflation of the resulting chromanones **2** using triflic anhydride in the presence of the proton sponge 2,6-di-*tert*butyl-4-methylpyridine (DTBMP). After activation of resin **6** by treating TfOH, eight different vinyl triflated intermediates **2** were immobilized on these activated resins **7** in the presence of 2,6-lutidine to afford intermediates **12** on solid supports (average loading level: ~0.9 mmol/g).

The various substituted aryl rings (R^3^) were introduced via palladium-mediated Suzuki coupling of aryl boronic acids. Among the many conditions tested in the solid-phase, the reaction condition with Pd(PPh_3_)_4_ and Na_2_CO_3_ in aqueous 1,4-dioxane displayed a robust chemical transformation of **3** with various substituted aryl boronic acids (18 different aryl boronic acids), resulting in high yields of the desired 3-substituted 2*H*-benzopyran resins **13**. Finally, the privileged benzopyrans **10** were produced by cleavage of resins **13** by using HF/pyridine in tetrahydrofuran (THF) and subsequent quenching with TMSOEt, and the resulting 144-member small-molecule collection was synthesized on a scale of 10–20 mg and their average purity was 87% without any purification steps.

**Scheme 3:**
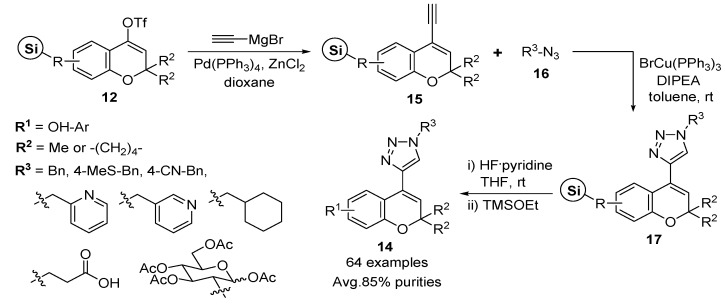
Solid-phase synthesis of 1,2,3-triazole-substituted 2*H*-benzopyrans **14** by Park *et al.* [51].

Additionally, the solid-phase synthesis of the mono-substituted 2*H*-benzopyran-attaching 1,2,3-triazole ring at position 4 was described by Park and co-workers [51]. For the solid-phase synthesis of 1,2,3-triazole-substituted 2*H*-benzopyrans **14**, terminal alkyne resins **15** were introduced to the vinyl triflate intermediates **12** on the solid support through a palladium-mediated Negishi-type cross-coupling reaction (Scheme 3) [51]. After Negishi-type alkynylation with ethynylmagnesium bromide, Pd(PPh_3_)_4_, and ZnCl_2_, the resulting terminal alkynyl moiety on the benzopyran core skeletons with azides **16** was subjected to regioselective Huisgen 1,3-dipolar [3 + 2] cycloaddition, namely, Click chemistry, in the presence of BrCu(PPh_3_)_3_ [52,53,54], a Cu-catalyst soluble in organic solvent, and *N*,*N*-diisopropylethylamine (DIPEA) to yield a new 1,2,3-triazole-substituted 2*H*-benzopyran resins **17** using solid-phase parallel synthesis. The eight different azides **16** were utilized for the Click chemistry and produced the novel 1,2,3-triazole-substituted 2*H*-benzopyrans **14** (64 examples) after HF/pyridine cleavage from the solid support and subsequent quenching with TMSOEt. The average purity, measured by liquid chromatography-mass spectrometry (LC-MS) analysis of the crude products, was 85%.

**Scheme 4:**
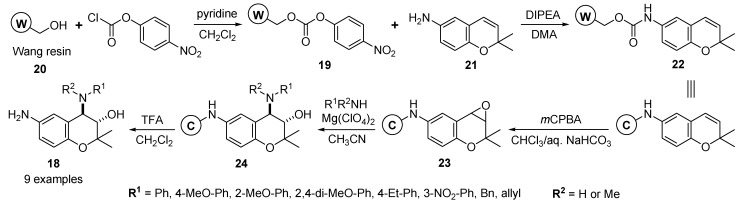
Solid-phase synthesis of 3-hydroxy-4-amino-substituted benzopyrans **18** by Gong and Yoo [55].

Gong and Yoo reported the solid-phase synthesis of 3-hydroxy-4-amino-substituted benzopyrans **18** via epoxide opening in a two-phase solvent system [55]. The reaction of 4-nitrophenyl carbonate resin **19**, which was formed by the reaction of Wang resin **20** and *p*-nitrophenyl chloroformate in CH_2_Cl_2_, with 6-amino-2,2-dimethyl chromene (**21**) and DIPEA in *N*,*N*-dimethylacetamide (DMA) afforded the carbamate resin **22** (Scheme 4).

After various solvent systems and oxidizing agents were examined to avoid the formation of *m*-chlorobenzoic acid-added adduct resin in the case of oxidation of resin **22** with *m*-chloroperbenzoic acid (*m*CPBA), the two-phase solvent system comprised of chloroform and saturated aqueous NaHCO_3_ with *m*CPBA afforded the epoxide resin **23**. The regioselective ring opening of the polymer-bounded epoxide **23** with nine amines produced the 3-hydroxy-4-amino-substituted benzopyran resins **24** in good overall yields without significant contamination of the by-products. The desired benzopyran derivatives **18** (9 examples) were finally liberated from the resin **24** using trifluoroacetic acid (TFA).

#### 2.1.3. Solid-Phase Synthesis of 6-Substituted Benzopyran Compounds

**Scheme 5:**
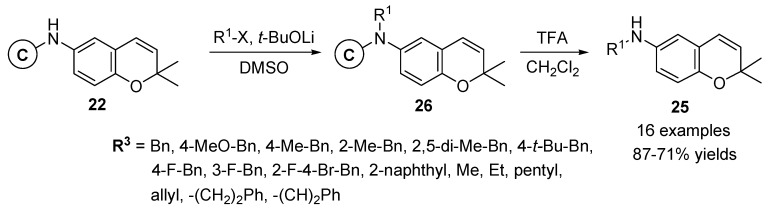
Solid-phase synthesis of 6-amino-substituted 2*H*-benzopyrans **25** by Gong *et al.* [56].

Solid-phase synthesis of 6-amino-substituted 2*H*-benzopyrans **25** was reported for the enlargement of diverse points in the benzopyran moiety by Gong *et al.* [56]. The *N*-alkylation [57,58] of the carbamate resin 22 [55] with alkyl halides and lithium *t*-butoxide in dimethyl sulfoxide (DMSO) introduced subsequently to various alkyl substituents in the 6-amino moiety of resins **26**. The desired 6-amino-substituted 2*H*-benzopyran products **25** (16 examples, 87–71% yields) were liberated from the resins **26** with TFA (Scheme 5).

#### 2.1.4. Solid-Phase Synthesis of 8-Substituted Benzopyran Compounds

**Scheme 6:**
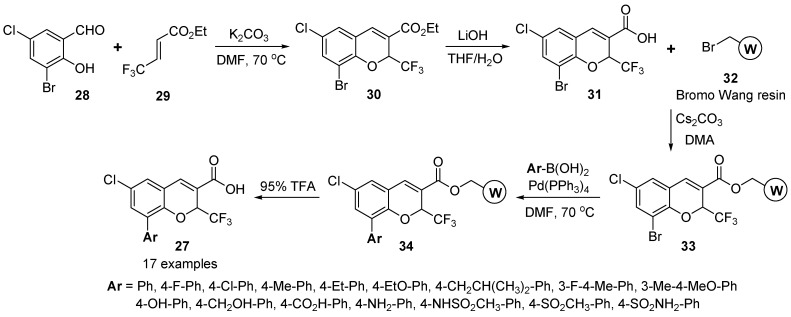
Solid-phase synthesis of 8-substituted 2*H*-benzopyrans **27** by Liao *et al.* [59].

The new lead SD-8381 (see Figure 1) with 2*H*-benzopyran was identified as a novel COX-2 inhibitor from in-house HTS [25,26]. The synthesis of SD-8381 derivatives **27** (8-substituted 2*H*-benzopyrans) was carried out on solid-phase parallel synthetic approach to find more potent COX-2 inhibitors by Liao *et al.* (Scheme 6) [59].

Aa a starting material, 3-bromo-5-chloro-2-hydroxybenzaldehyde (**28**) was reacted with ethyl trifluoro-methylcrotonate (**29**) in the presence of K_2_CO_3_ and *N*,*N*-dimethylformamide (DMF) under nitrogen at 70 °C (Scheme 6). The resulting 2*H*-benzopyran intermediate **30** was produced in 90% yield. After hydrolysis of ester **30** with aqueous lithium hydroxide in THF, acid **31** with an attached point was obtained in 80% yield. The benzopyran acid **31** was then loaded on bromo Wang resin **32** [4-(bromomethyl)phenoxymethyl-polystyrene] in the presence of cesium carbonate/DMA at 60 °C. Subsequently the bromo group on benzopyran **33** was converted into various aromatic substituents via palladium-mediated Suzuki coupling catalyzed by Pd(PPh_3_)_4_ with a diverse set of aromatic boronic acids [60]. The desired 8-substituted 2*H*-benzopyrnas **27** (17 examples) were cleaved from resin **34** with 95% TFA in CH_2_Cl_2_ using triisopropylsilane as scavenger. A few analogs of 8-substituted 2*H*-benzopyran **27** (Ar = 4-Et-Ph and 3-Me-4-MeO-Ph) were used for further investigation *in vivo*.

### 2.2. Solid-Phase Synthesis of Disubstituted Benzopyran Compounds

#### 2.2.1. Solid-phase Synthesis of 2,3-Disubstituted Benzopyran Compounds

**Scheme 7:**
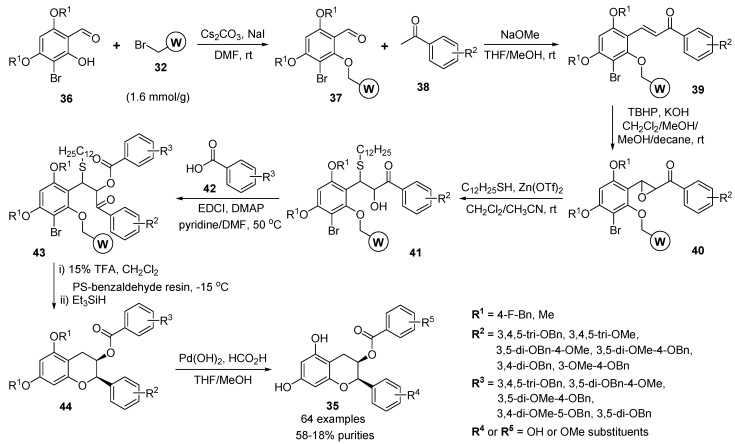
Solid-phase synthesis of 2,3-disubstituted benzopyrans **35** by Takahashi *et al.* [61,62].

Takahashi and co-workers described the efficient solid-phase synthesis of EGCG (see Figure 1) and the combinatorial synthesis of protected methylated epicatechin derivatives **35** (2,3-disubstituted benzopyran derivatives) (Scheme 7) [61,62].

The solid-phase synthetic strategy of 2,3-disubstituted benzopyrans **35** began with the treatment of the aldehyde **36** with bromo Wang resin **32** (1.6 mmol/g) to provide the aldehyde resins **37** [63]. The treatment of the aldehyde resins **37** with the methyl ketones **38** under NaOMe basic conditions provided the solid-supported enone **39**, which underwent epoxidation with *t*BuOOH to give the solid-supported epoxide **40**. The regioselective epoxide-ring opening [64] of **40** with 1-dodecanethiol in the presence of Zn(OTf)_2_ proceeded without cleavage of the Wang linker to afford the solid-supported a-hydroxyketone **41**, the acylation of which with benzoic acids (**42**) then gave the precursor **43** for reductive cyclization. The exposure of 15%TFA in CH_2_Cl_2_ in the presence of a PS–benzaldehyde resin followed by the addition of triethylsilane promoted the cleavage of the Wang linker, reduction of the sulfide and the bromide, and reductive etherification to provide the protected 2,3-disubstituted benzopyrans **44**. Finally, the 2,3-disubstituted benzopyran derivatives **44** were deprotected by conventional hydrogenolysis in the solution-phase by using a palladium catalyst to provide EGCG derivatives **35** (64 examples).

The two aldehydes **36**, six ketones **38**, and five carboxylic acids **42** were used as building blocks for the synthesis of 60 members of 2,3-disubstituted benzopyran library. The purity of the library was estimated by LC-MS analysis (58–15% purities). The growth-inhibitory effects of the resulting library compounds were examined [62]. Most of the 7-OMe derivatives exhibited biological activity comparable to that of the naturally occurring EGCG.

#### 2.2.2. Solid-Phase Synthesis of 2,6-Disubstituted Benzopyran Compounds

**Scheme 8:**
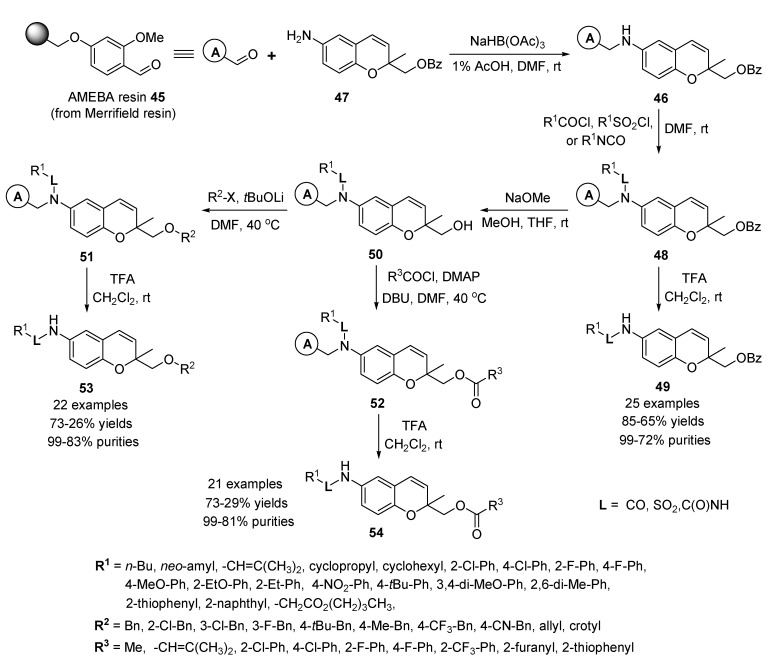
Solid-phase synthesis of 2,6-difunctionalized benzopyrans **49**, **53**, and **547** by Gong *et al.* [65].

Gong and co-workers reported the construction of a 2,6-difunctionalized 2*H*-benzopyran library of 1,200 analogues by using the solid-phase protocols [65]. An alternative linker-based synthetic strategy was developed because of a restriction that a carbamate linker based solid-phase synthetic pathway to generate a substituted benzopyran library (**18** and **25**) could not be introduced at the 2-position of the benzopyran system by using strong bases. In the strategy, acid sensitive methoxy benzaldehyde (AMEBA) resin **45** [2-(4-formyl-3-methoxyphenoxy)ethyl polystyrene from Merrifield resin] [66] was selected as the polymer support since the secondary amino group, resulting from reductive amination, should be highly reactive towards various alkyl halides, acid halides, isocyanates, and sulfonyl chlorides (Scheme 8). Moreover, the final products should be readily cleaved from the support by using dilute TFA solutions [67,68]. In the first step of the sequence, 6-aminobenzopyran resin **46** was prepared by reaction of AMEBA resin **45** with 6-aminobenzopyran **47** [69] under reductive amination conditions [70] [NaBH(OAc)_3_ in DMF containing 1% acetic acid].

In the first-generation diversification step, the secondary amine group in **46** was transformed into the amide, sulfonamide, or urea groups in resin **48** by respective reactions with acid chlorides, sulfonyl chlorides, and isocyanates in the presence of triethylamine in DMF. To confirm the product formation, resin **48** was treated with 20% TFA in CH_2_Cl_2_ to give 6-amino-substuted 2*H*-benzopyran **49** (25 examples, 85–65% yields, and 99–72% purities).

For the purpose of second-generation diversity, resins **50** containing a free primary hydroxyl group were prepared by reaction of resins **48** with NaOMe in MeOH/THF at room temperature [71]. Functionalization of the hydroxyl groups in resins **50** was promoted by reactions with alkyl halides and acid chlorides to generate respective 2,6-difuctionalized 2*H*-benzopyran resins **51** with an ether-substituent and **52** with an ester-substituent at position 2 in the 2*H*-benzopyran moiety. Alkylation reactions of **50** were carried out in the presence of lithium *tert*-butoxide in DMF and took place smoothly to yield the corresponding ethers. Subsequent treatment of the resins **51** with 20% TFA in CH_2_Cl_2_ produced the desired 2,6-difuctionalized 2*H*-benzopyran derivatives **53** with an ether-substituent (the representative 22 examples, 73–26% yields, and 99–83% purities) in high four-step overall yields from resin **46**. The ester-containing resins **52** were prepared by treatment of resins **50** with various acid chlorides in the presence of DBU and 4-dimethylaminopyridine (DMAP) in DMF. To confirm product formation, the resins **52** were treated with 20% TFA in CH_2_Cl_2_ to yield the desired 2,6-difuctionalized 2*H*-benzopyrans **54** with an ester-substituent (the representative 21 examples, 73–29% yields, and 99–81% purities).

**Scheme 9:**
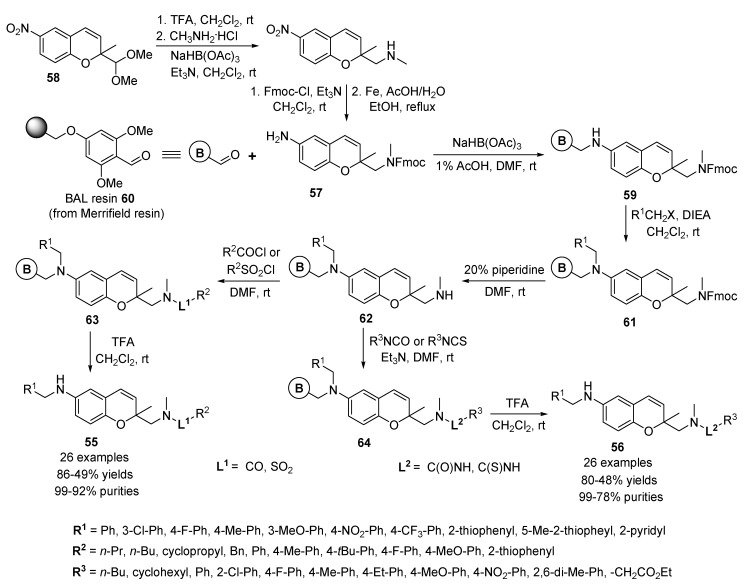
Solid-phase synthesis of 6-alkylamino-2-(functionalized-aminomethyl)-2*H*-benzopyrans **55** and **56** by Gong *et al.* [72].

Also, Gong and co-workers developed the solid-phase parallel synthesis of the additional 2,6-difunctionalized 2*H*-benzopyrans **55** and **56**, which provided a 2,000-member library of novel 6-alkylamino-2-(functionalized-aminomethyl)-2*H*-benzopyrans [72]. The overall synthetic strategy used to prepare the target 2*H*-benzopyran analogues **55** and **56** is outlined in Scheme 9. The Fmoc-protected 2*H*-benzopyran amine **57** was prepared from 2-dimethoxymethyl-2-methyl-2*H*-1-benzopyran **58** [31] using the reaction sequences by acetal deprotection, reductive amination with methylamine, secondary amine protection with Fmoc-Cl, and reduction of nitro-group.

In the parallel solid-phase protocol (Scheme 9), 6-aminobenzopyran resin **59** was prepared from the backbone amide linker (BAL) resin **60** [73,74,75] by reaction with 6-aminobenzopyran **57** under reductive amination conditions with NaBH(OAc)_3_ [70].

In the first-generation diversification step, resin **61**, containing a secondary amine group, was reacted with alkyl halides in the presence of diisopropylethylamine (DIEA) in CH_2_Cl_2_. For second-generation diversification, resins **61** containing a secondary amino group were prepared by removal of Fmoc on resins **61** with 20% piperidine in DMF. Functionalization of the secondary amine groups on resins **62** is promoted by reaction with various electrophiles, including acid chlorides, sulfonyl chlorides, isocyanates, and isothiocyanates. This leads to the generation of the respective amide, sulfonamide, urea and thiourea derivatives. Further confirmation of product formation was accomplished by treatment of resins **63** and **64** with 20% TFA in CH_2_Cl_2_ and by characterization of the liberated 2,6-difunctionalized 2*H*-benzopyrans **11** and **12** with 2-functionalized-aminomethyl group.

Lipinski’s rule [76] and similar formulations [77,78] serve as guidelines to estimate the physicochemical properties of the 2,000-member library of 6-alkylamino-2-(functionalized-aminomethyl)-2*H*-1-benzopyran derivatives **55** and **56**. Most of the key parameters for members of the library fall within the range of those predicted for reasonable oral bioavailable drugs by using the commonly known guidelines.

The drug-like small molecule library with variously substituted benzopyrans was constructed by Gong and co-workers [79,80]. An orally active 5-LO [81,82,83,84,85,86,87] inhibitor KRH-102140 [30,31] with the benzopyran moiety (see Figure 1) was discovered as a lead compound on the drug discovery program via HTS of in-house small molecule library by Gong *et al.* [88]. Also, KRH-102140 showed hypoxia-inducible factor (HIF)-1α [89,90,91,92,93] inhibitory activities [94].

On the basis of the biological activities of the benzopyran moiety with KRH-102140, Gong and co-workers demonstrated the solid-phase synthesis of a 222-number library of 2,6-difunctionalized 2*H*-benzopyran **65** and **66** for the lead optimization as a 5-LO inhibitor [80].

**Scheme 10:**
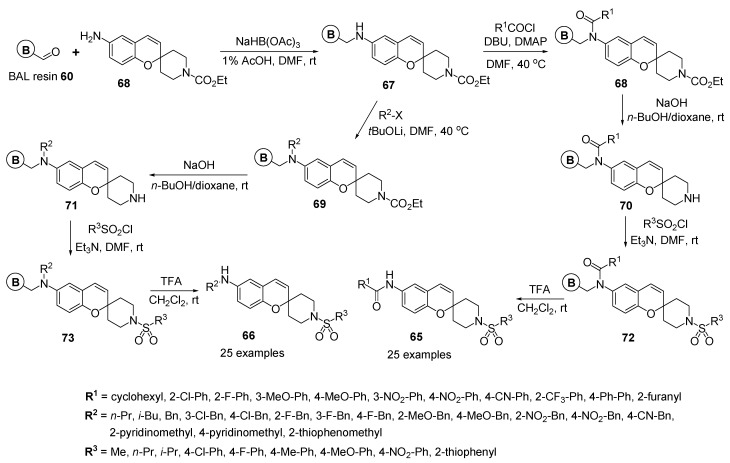
Solid-phase synthesis of 6-amido- and 6-amino-substituted-2-functionalized benzopyrans **65** and **66** by Gong *et al.* [80].

As shown in Scheme 10, resin-bounded spirobenzopyran **67** was prepared by reaction of BAL resin **60** with *N*-[ethylcarbamate-spiro(2*H*-1-benzopyran-2,4-piperidine)-6-yl]amine **68**, which was synthesized by general manipulations, under reductive amination conditions. In the first generation diversification step, the secondary amine group on resin **67** was transformed into the tertiary amine and amide on benzopyran resins **68** and **69** at position 6 by the reactions with various acid chlorides and alkyl halides in the presence of bases, respectively.

After the carbamate deprotection in a spiro-ring of benzopyran resins **68** and **69** by hydrolysis reactions, the secondary piperidine amines on the 6-amino- or 6-amido-substituted 2*H*-benzopyran resins **70** and **71** were converted by reactions with various sulfonyl chlorides to generate respective 6-amino-substituted amide resins **72** and 6-amino-substituted resins **73** for the introduction of second generation diversity.

Finally, the liberation of solid support on the resins **72** and **73** with 20% TFA in CH_2_Cl_2_ gave the desired drug-like 2-functionalized-6-amido-substituted 2*H*-benzopyran derivatives **65** (the representative 25 examples) and 2-functionalized-6-amino-substituted 2*H*-benzopyran derivatives **66** (the representative 25 examples), respectively.

In general, the goal of a drug discovery process is to synthesize chemical entities which are orally bioavailable; *i.e.* they possess physiological properties that allow them to be absorbed into the gastrointestinal system. Lipinski’s Rule [76] and similar formulations [77,78] served as guidelines to estimate the physicochemical properties of the synthesized 222-member library of 6-amido- and 6-amino-substituted-2-functionalized benzopyrans **65** and **66**, respectively [80].

### 2.3. Solid-Phase Synthesis of Trisubstituted Benzopyran Compounds

#### 2.3.1. Solid-Phase Synthesis of 2,4,6-Trisubstituted Benzopyran Compounds

**Scheme 11:**
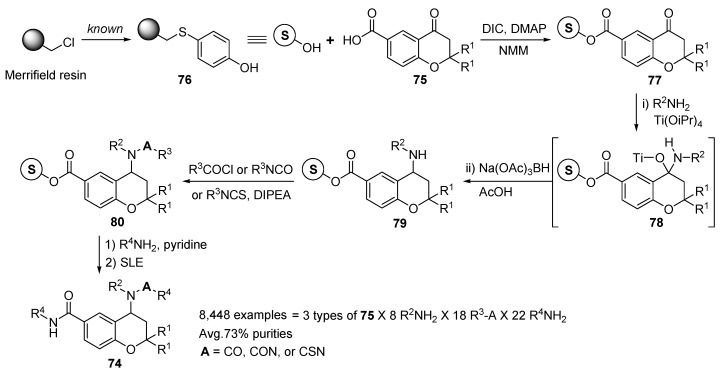
Solid-phase synthesis of 2,4,6-trisubstituted benzopyrans **74** by Breitenbucher and Hui [95].

Breitenbucher and Hui developed the titanium-mediated reductive amination procedure for the practical solid-phase synthesis of 2,4,6-trisubstituted benzopyran derivatives **74** (2,4,6-trisubstituted chromane) [95]. The three different 6-carboxybenzopyran-4-one scaffolds **75** [96] was attached to Merrifield/hydroxythiophenol resin **76** via diisopropylcarbodiimide (DIC) coupling, to provide benzopyranone resins **77** (Scheme 11). Treatment of resin **77** with Ti(O*i*Pr)_4_ and a primary amine (eight R^1^NH_2_) in toluene provided a tetrahedral intermediate resin **78**. Na(OAc)_3_BH was then added to the reaction to effect reduction to the amine on resin, followed by washings to afford 4-amino-benzpyran resins **79**.

Acylation (16 acylating agents) of the 24 different resins **79** was then performed by addition of either an isocyanate, or acid chlodde to afford 4-disubstituted benzopyran resins **80**. Cleavage from support was accomplished by treatment with 4 equivalents of an amine (22 amines) in pyridine at room temperature. The resulting library products **74** (8,448 spatially separated benzopyrans) were then concentrated and subjected to supported liquid extraction (SLE) [97,98] to remove the excess cleaving amines from the products. Their average purity of samples measured by LC-MS analysis of the crude products was around 73%.

#### 2.3.2. Solid-Phase Synthesis of 3,4,6-Trisubstituted Benzopyran Compounds

**Scheme 12:**
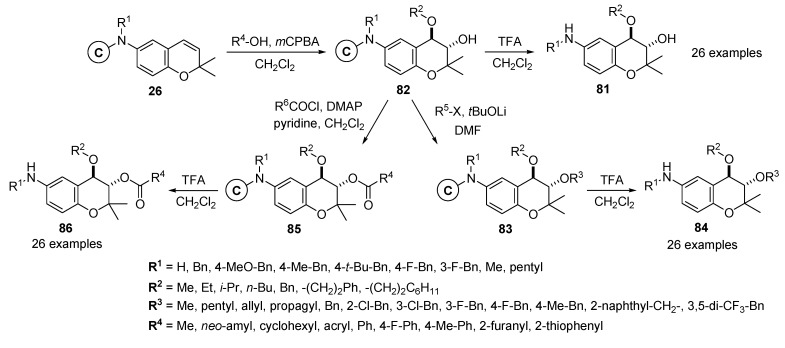
Solid-phase synthesis of 3,4,6-trisubstituted benzopyrans **84** and **86** by Gong *et al.* [99].

Gong and co-workers described the construction of a 3,4,6-trisubstituted benzopyran library of 2,000 analogues using consecutive nucleophilic addition *via m-*CPBA epoxidation on solid support [99,100]. Various reaction conditions were examined to find a condition of the nucleophilic alcohol addition at an epoxide on resin and completion of 3-hydroxy-4-alkoxy-6-amino-substituted benzopyrans **81** on solid support. The desired 3-hydroxy-4-alkoxy-benzopyran resins **82** were obtained by the consecutive nucleophilic alcohol addition reactions of resins **26** with nucleophiles, immediately followed by *m-*CPBA epoxidation. Finally, the cleavage of resins **82** with 25% TFA in CH_2_Cl_2_ produced the target 3-hydroxy-4-alkoxy-6-amino-substituted benzopyrans **81** (26 examples) without significant contamination of by-products (Scheme 12).

After confirmation of the consecutive regioselective nucleophilic addition *via m-*CPBA epoxidation on solid support, the 3-hydroxyl benzopyran resins **82** were scrutinized for enlargement of diverse points in the benzopyran moiety. The reactions with alkyl halides and acid chloride were examined to generate ethers and esters in 3-hydroxyl benzopyran resins **82**. To prepare the ether-type at position 4 in resins **83**, the resins **82** were treated with various alkyl and benzyl halides in the presence of lithium *t*-butoxide in DMF. The reaction proceeded nicely to provide polymer-bounded benzopyrans **83** with an ether-substituent, and subsequent treatment of the resins **83** with 25% TFA in CH_2_Cl_2_ produced the desired 3,4,6-trisubstituted benzopyrans **84** with an ether-substituent (the representative 26 examples) in good four-step overall yields.

For the preparation of esters at position 4 in the benzopyran moiety, the resins **82** were treated with various acid chlorides with pyridine and DMAP as bases in CH_2_Cl_2_, to produce the benzopyran resins **85** with an ester-substituent, which were again treated with 25% TFA in CH_2_Cl_2_ h to give the desired 3,4,6-trisubstituted benzopyrans **86** with ester-substituent (26 representative examples) in good four-step overall yields. The obtained 3,4,6-trisubstituted benzopyrans **84** with an ether-substituent were identified as prolyl 4-hydroxylase inhibitors via a screening process using HSC-T6 and LI 90 cells that express an immortalized rat hepatic stellate cell line and as part of a test of the type I collagen contents employing the ELISA method [100].

## 3. Solid-Phase Synthesis of Additional Cycle-Fused Benzopyran Compounds

### 3.1. Solid-Phase Synthesis of Tricyclic Benzopyran Compounds

#### 3.1.1. Solid-Phase Synthesis of Isoxazole-Fused Benzopyran Compounds

Collins and co-workers developed the solid-phase synthesis of isoxazole-fused benzopyrans (benzopyranoisoxazoles) **87** as potential steroid mimetic templates using intramolecular 1,3-dipolar cycloaddition with a tethered alkyne [101].

**Scheme 13:**
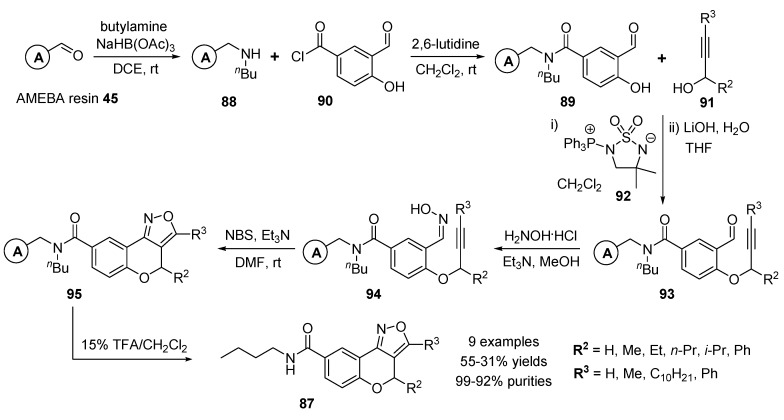
Solid-phase synthesis of isoxazole-fused benzopyrans **87** by Collins and co-workers [101].

The reductive amination reaction of the commercially available AMEBA [66] resin **45** (2-(4-formyl-3-methoxyphenoxy)ethyl polystyrene) and butylamine with sodium triacetoxyborohydride gave polymer-bounded secondary amine **88** (Scheme 13). 3-Formyl-4-hydroxybenzoyl moiety loaded resin **89** was introduced by amide formation of amine resin **88** and 3-formyl-4-hydroxybenzoyl chloride (**90**) in the presence of 2,6-lutidine. The various Mitsunobu reactions [102,103,104,105,106] were explored for a polymer-bounded aldoxime containing a phenol-tethered alkyne **94**. A Mitsunobu reaction between polymer-bounded phenol **89** and propargyl alcohol **91** with R^2^ and R^3^ using sulfonamide betaine **92** gave disubstituted resins **93**. The isoxzole precursor resins **94** with aldoxime and alkyne were obtained upon treatment of aldehyde resins **93** with hydroxylamine hydrochloride and triethylamine.

An intramolecular 1,3-dipolar cycloaddition of **94** with NBS and Et_3_N in DMF generated polymer-bounded benzopyranoisoxazoles **95**. After cleavage with 15% TFA in CH_2_Cl_2_, the target benzopyranoisoxazoles **87** (9 examples) were obtained.

#### 3.1.2. Solid-Phase Synthesis of Pyrazole-Fused Benzopyran Compounds

**Scheme 14:**
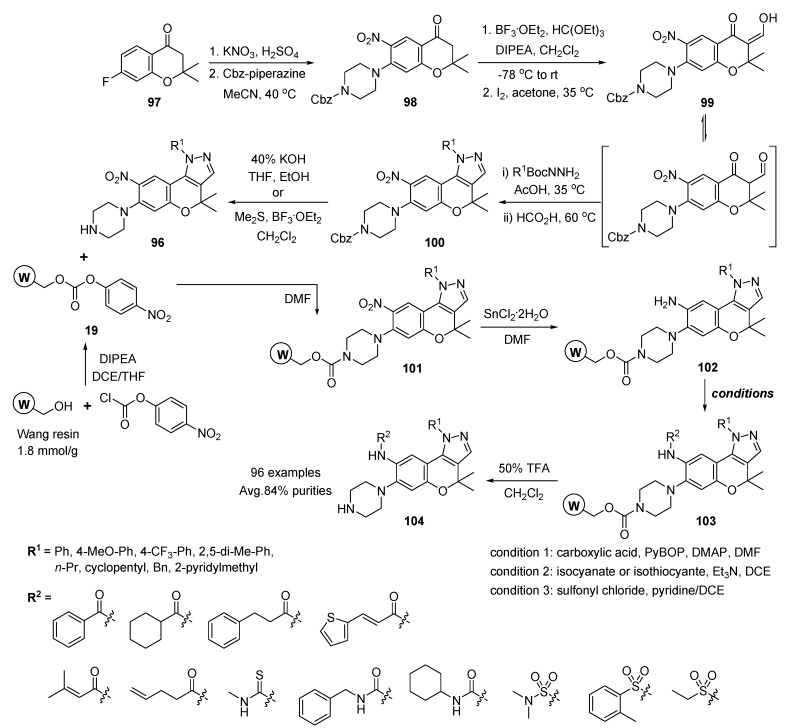
Solid-phase synthesis of pyrazole-fused benzopyrans **104** by Park *et al.* [107].

Park and co-workers developed the solid-phase construction of a pyrazole-fused benzopyran (benzopyranopyrazole) library with the recombination of privileged benzopyran and pyrazole structures [107]. The solid-phase synthesis was started from the functionalized benzopyranopyrazoles **96** (Scheme 14). A 7-fluoro-2,2-dimethyl-2,3-dihydrochromen-4-one **97** underwent nitration with potassium nitrate to introduce the aniline moiety in a masked form, followed by nucleophilic aromatic substitution of monoprotected Cbz-piperazine at the fluoride position because of its rich body of biological effects. The resulting chromenone **98** was converted to s-*cis* enone **99** by the sequent reactions of acetal formation [108,109,110] with triethyl orthoformate in high yield (93%) and I_2_-catalyzed acetal deprotection [111,112,113,114,115,116,117] (84% yield). In the case of an electron-deficient chromenone **98**, the acidic proton at the C-3 position was readily removed by treatment with sodium methoxide (the reaction condition for hydroxysubstituted s-*cis* enone **99**), which led to the decomposition of benzopyran itself.

The regioselective synthesis of benzopyranopyrazole derivatives **100** was achieved by the condensation of a b-keto aldehyde with mono-substituted hydrazine (R^1^BocNNH_2_) in AcOH [108,109]. The Cbz protection group on the piperazine moiety was removed from benzopyranopyrazole **96** by 40% KOH or dimethyl sulfate and BF_3_·OEt_2_ [118] for the immobilization of the piperazinyl secondary amine **96** on a solid support.

As shown in Scheme 14, Wang resin was activated with *p*-nitrophenylchloroformate in the presence of DIPEA, followed by the loading of the piperazinyl secondary amine **96** on the solid support. The nitro group on polymer-bounded intermediates **101** was reduced with tin chloride dihydrate in DMF. The resulting aniline resins **102** were subsequently diversified with a set of 12 building blocks (six carboxylic acids, one isothiocyanate, two isocyanates, and three sulfonyl chlorides) identical to that used for the modification at the R^2^ position. The final cleavage step with resins **103** was performed under 50% TFA in dichloromethane to liberate various benzopyranopyrazoles **104** (96 examples). Overall, the average purity of the final 96 benzopyranopyrazoles **104** with R^1^ and R^2^ diversification was 84%.

#### 3.1.3. Solid-Phase Synthesis of Six-Membered Ring-Fused Benzopyran Compounds

**Scheme 15:**
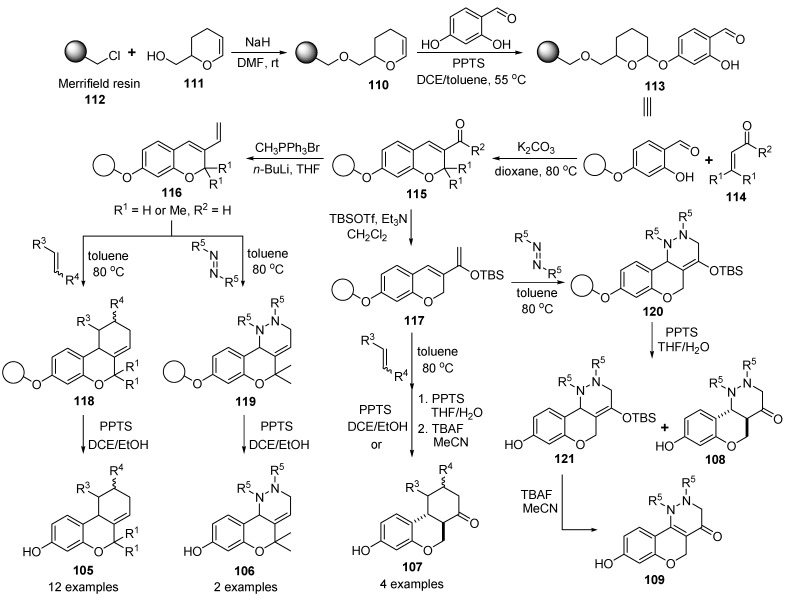
Solid-phase synthesis of tricyclic benzopyrans **105**–**109** by Bräse and Kapeller [119].

A novel solid-phase synthetic approach toward tricyclic benzopyrans **105**–**109** (cannabinoid derivatives) is described by Bräse and Kapeller [119]. The synthetic approach for the solid-phase synthesis of tricyclic benzopyrans **105**–**109** was to immobilize the commercially available 4-hydroxysalicylaldehyde (4-HSA) with Ellman’s acid-labile DHP-linker **110** [120,121], which was prepared by etherification of a 3,4-dihydro-2*H*-pyran-2-methanol (**111**) and Merrifield resin **112** [122] (0.99 mmol/g). At first, polymer **110** was treated with 4-HSA/PPTS (pyridinium *p*-toluenesulfonate) to give polymer-bounded aldehyde **113** (Scheme 15).

**Figure 4:**
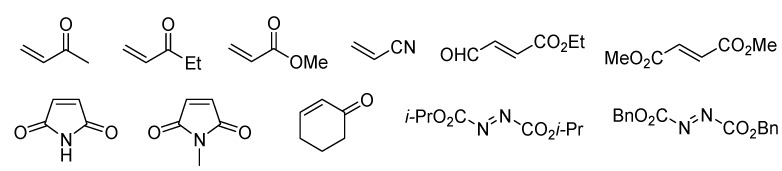
Diverse dienophile reagents for the Diels-Alder reaction.

To generate the benzopyran core structure, the domino oxa-Michael-aldol (DOMA) condensation [123,124,125] for the resin **113** and a,b-unsaturated carbonyl moieties **114** as Michael acceptors gave polymer-bounded benzopyrans **115** by employing K_2_CO_3_ in 1,4-dioxane at 80 °C. The next step for a diene moiety was either Wittig reaction with CH_3_PPh_3_Br to **116** or TBS-enol ether formation yielding **117**. For the Diels-Alder reaction, a thermal condition at 80 °C was chosen, as Lewis or Brønsted acid catalysis would lead to concomitant cleavage from the resin. The Diels-Alder reaction of diene resins **116** and various dienophiles (see Figure 4) gave tricyclic benzopyran resins **118** and **119** in this thermal condition. After cleavage of resins **118** and **119** with PPTS in DCE/EtOH, the desired tricyclic benzopyran derivatives **105** and **106** (14 examples, 95–11% yields) were obtained. Also, enol ether resin **117** was subjected to the Diels-Alder reaction. The resulting tricyclic benzopyran resins **120** with enol ether were at first cleaved with PPTS in DCE/EtOH giving ketones **107** directly under concomitant loss of the TBS-group. When switching the solvent to THF/water, but the TBS group was only partially cleaved-off, mainly giving enol-ethers **121**. Further treatment with TBAF in acetonitrile finally liberated tricycles **107** (4 examples, 51–10% yields) in all cases except for **108** (R^5^ = CO_2_*i*Pr), where side-product **109** was formed exclusively.

**Scheme 16:**
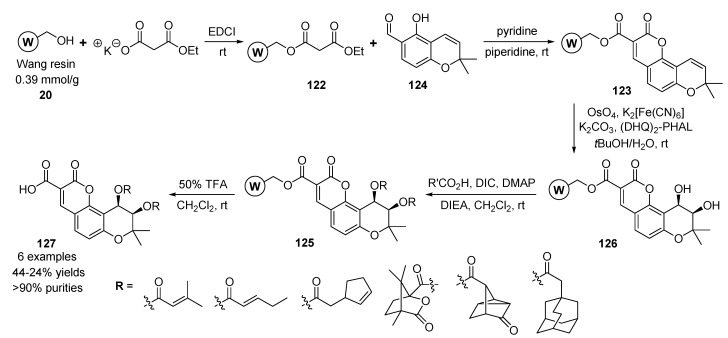
Asymmetric solid-phase synthesis of tricyclic benzopyrans **127** by Lee *et al.* [126,127].

Lee and co-workers described the asymmetric solid-phase parallel synthesis of (3'*R*,4'*R*)-di-*O*-*cis*-acyl 3-carboxyl khellactones (tricyclic benzopyrans) as potent anti-HIV agents [126,127]. The strategy was begun with ethyl malonate bound resin **122**, which was introduced by a reaction of Wang resin **20** and ethyl potassium malonate (Scheme 16). The resin-bounded tricyclic benzopyran **123** was prepared by a Knoevenagel condensation [128,129,130,131] between ethyl malonate resin **122** and *o*-hydroxy-arylaldehyde **124** in pyridine and piperidine. The *o*-hydroxyarylaldehyde **124** was prepared from an acetyl acetaldehyde dimethyl acetal using the reaction sequences by Grignard addition, nucleophilic substitution of 2,4-dihydroxybenzoylaldehyde, and regiospecific aromatic cyclization.

The polymer-bounded 3,4-diacyl-substitued tricyclic benzopyrans **125** were obtained by acylation with various carboxylic acids and an optically active *cis*-diol resin **126** from the Sharpless asymmetric dihydroxylation (AD) reaction of resin **123**. The treatment of diacyl resins **125** with TFA in CH_2_Cl_2_ gave the desired tricyclic benzopyran derivatives **127** (6 examples, 44–24% yields, and >90% purities).

### 3.2. Solid-Phase Synthesis of Polycyclic Benzopyran Compounds

**Scheme 17:**
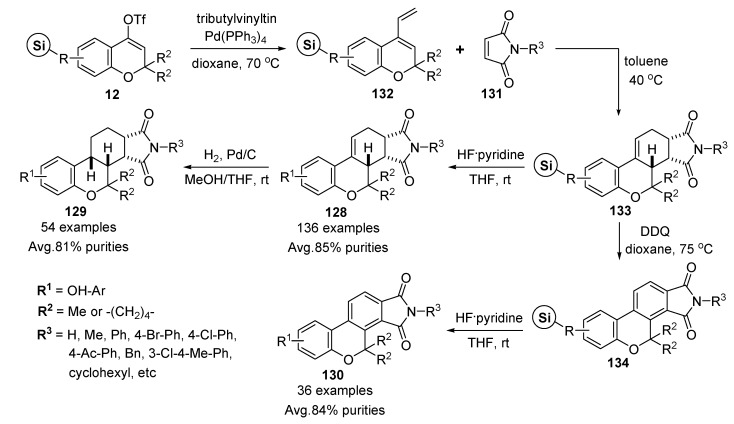
Solid-phase synthesis of polycyclic benzopyrans **128**–**130** by Park *et al.* [51].

Novel polycyclic scaffolds **128**–**130** containing the benzopyran moiety with variable substituents were described by Park* et al.* [51]. The excellent *endo*-selective Diels-Alder reaction with dienophiles **131** (17 substituted maleimides) and solid-supported dienes **132** (8 resins from **2**, see Scheme 2 and Figure 3), which were derived from palladium-mediated Stille-type vinylation on vinyl triflate intermediate **12**, gave benzopyran-containing polycycloheterocyclic resins **133** (Scheme 17). After HF/pyridine cleavage of resins **133** and subsequent quenching with TMSOEt, diastereomerically enriched novel tricyclic benzopyran derivatives **128** were obtained on a scale of 10 mg each (136 examples). Their average purity measured by LC-MS analysis of the crude products, was around 85%.

To expand the molecular diversity of the small-molecule library, novel polycyclic benzopyran derivatives **128** were transformed to discrete core skeletons **129** and **130**, using chemical transformations such as Pd/C-based diastereoselective hydrogenation of **128** by the library-to-library approach and the sequence reaction of 2,3-dichloro-5,6-dicyanobenzoquinone (DDQ)-mediated aromatization of resins **133** and liberation of resins **134**, respectively. The average purity of **129** (54 examples), which obtained from **12** without further purification, was about 81%, and that of **130** (36 examples) measured by LC-MS analysis of the crude products, was 84%.

The shape of the resulting core skeleton **129** is structurally discrete and more concave than that of its precursor **128** because of the conversion at the monoene site of *sp*^2^ carbon to *sp*^3^ carbon. Compared to heterogeneous hydrogenation, which introduces the *sp*^3^ carbon center in an asymmetric fashion, the aromatization using DDQ can remove the existing stereogenic carbon centers of the monoene precursor **128** and provide a new flatter core skeleton **130**.

## 4. Solid-Phase Synthesis of Miscellaneous Benzopyran Compounds

**Scheme 18:**
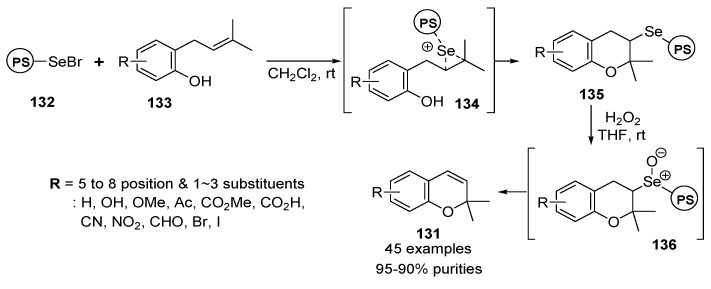
Solid-phase synthesis of benzopyrans **131** by Nicolau *et al.* [132,133,134,135,136,137,138].

The selenium-mediated solid-phase syntheses of various 2,2-dimethylbenzopyran derivatives **131** were published by Nicolau *et al.* [132,133,134,135,136,137,138]. The solid-phase synthetic strategy started from the treatment of selenyl bromide resin **132** [139,140] with a three-fold excess of various *ortho*-prenylated phenols **133** in CH_2_Cl_2_. The dihydrobenzopyran resins **135** were produced via a 5-*endo*-*trig* cycloaddition of **134**. The desired 2*H*-benzopyrans **131** (45 examples) were obtained in high yields with high purities by oxidation to selenium oxide with H_2_O_2_ and *syn*-elimination of intermediate resin **136** in a traceless manner (Scheme 18).

**Scheme 19:**
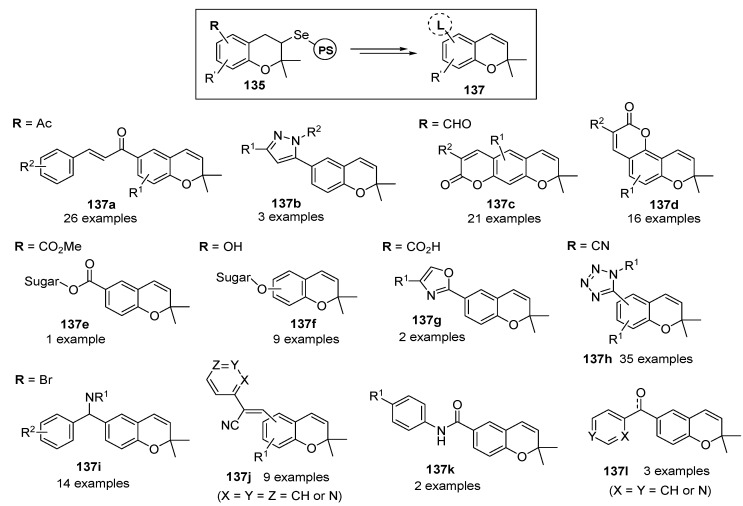
Benzopyran-based privileged structures from 2-selenodihydrobenzopyran resins **135** by Nicolau *et al.* [132,133,134,135,136,137,138].

After preliminary studies, their utility was demonstrated for the syntheses of the benzopyran-based privileged structures **137a**–**l** (natural benzopyrans, multi-substituted and polycyclic benzopyrans, and so on) from polymer-bounded 2-selenodihydrobenzopyrans **135** (Scheme 19).

The acquired polymer-bounded benzopyrans **135** were subsequently used as scaffolds in the synthesis of several benzopyran-based combinatorial libraries (for example, a 52-member library [137] aimed at the development of new NADH/ubiquinone oxidoreductase inhibitors, and a 10,000-member library [135] constructed by directed split-and-pool chemistry).

## 5. Summary

The combinatorial synthesis of drug-like small organic molecules plays a significant role in the area of drug discovery. Especially, the various natural and artificial benzopyran compounds as bioactive molecules have proven to be broadly useful as therapeutic agents because of their high degree of structural diversity. In this respect, many synthetic methods have been developed for fabricating the privileged benzopyran structures with drug-like properties by using solid-phase synthetic strategies. In this article, we have introduced the preparation of diverse and drug-like benzopyrans as substituted benzopyrans, additional cycle-fused benzopyrans, and their related compounds. Further studies in this area are underway and the various strategies for syntheses of benzopyran derivatives on solid support will be reported for medicinal chemistry and drug discovery.
